# Acute Flares of Knee Osteoarthritis (the ACT-FLARE Study): Protocol for a Web-Based Case-Crossover Study in Community-Dwelling Adults

**DOI:** 10.2196/13428

**Published:** 2019-04-22

**Authors:** Martin J Thomas, Trishna Rathod-Mistry, Stephen Harper, Emma L Parry, Christopher Pope, Tuhina Neogi, George Peat

**Affiliations:** 1 Primary Care Centre Versus Arthritis Research Institute for Primary Care and Health Sciences Keele University Staffordshire United Kingdom; 2 Haywood Academic Rheumatology Centre Haywood Hospital Midlands Partnership NHS Foundation Trust Staffordshire United Kingdom; 3 Keele Clinical Trials Unit Keele University Staffordshire United Kingdom; 4 Section of Rheumatology Department of Medicine Boston University School of Medicine Boston, MA United States

**Keywords:** knee, osteoarthritis, flare-up, Web-based study, case-crossover study

## Abstract

**Background:**

The cardinal feature of osteoarthritis (OA) is pain. Although heterogeneity in pain and function have been demonstrated in the long-term course of OA, the more proximate determinants of acute flare-ups remain less clear. How short-term intermittent or transient exposures trigger acute flare-ups has important implications for effective and sustainable self-management strategies.

**Objective:**

The primary objective of this study is to identify potential triggers of acute flares in knee OA. Secondary objectives are to determine their course and consequences and describe high-risk participant profiles.

**Methods:**

We carried out a Web-based case-crossover study. This study aims to recruit 620 community-dwelling adults aged ≥40 years, resident in England, and who have knee pain, with or without a recorded diagnosis of knee OA, and no preexisting diagnosis of inflammatory arthropathy. Participants will be recruited via 3 routes: (1) general practice registers, (2) offline community advertisement, and (3) online social media advertisement. By using questionnaires comparing periods before participants’ self-reported flare-up episodes (hazard periods) with periods during the study when their knee OA symptoms are stable (control periods), triggers preceding flare-ups will be identified and examined using conditional logistic regression. Time-to-resolution of flare-up will be examined by monitoring people’s daily pain, bothersomeness, and medication usage until the participant reports when their flare-up episode ends. Rates of flare-ups will be examined across different participant and flare characteristics using regression models to identify high-risk participant profiles. A study-specific Patient Advisory Group (PAG) is providing suggestion, input, and ongoing support for all stages of the research process.

**Results:**

Participant recruitment opened in July 2018 and is anticipated to continue for 6 months. The study results will be disseminated through a number of channels, including relevant national or international conferences and peer-reviewed publication in a medical journal, via advocacy or charity organizations, such as Versus Arthritis and across social media. Findings will be fed back to members of our PAG, study participants, and clinicians from participating primary care general practices. The PAG will also take an active role in the overall dissemination strategy.

**Conclusions:**

This study will provide empirical evidence to help patients identify common knee OA flare triggers and provide health care professionals with questions to identify patients at most risk of frequent flare-ups.

**International Registered Report Identifier (IRRID):**

DERR1-10.2196/13428

## Introduction

Pain caused by osteoarthritis (OA) is a major cause of functional limitation and disability worldwide [1]. The course of OA pain and functional limitation is heterogeneous among people [2-8], and within different long-term trajectories, there is evidence of substantial within-person variability over time. Shorter episodes of more severe pain (acute flare-ups) deserve further investigation for a number of reasons: (1) unpredictable episodes of severe pain are distressing and disabling in themselves and become more common in the late stages of OA [9], (2) they may disrupt patterns of healthy behavior that serve to reduce the risk of OA progression (eg, weight control, keeping active, and reducing sedentary time) [10], and (3) episodic flare-ups may herald (or may even cause) a transition to less favorable long-term trajectory.

Evidence of short-term daily impact of flare-ups of knee pain is emerging internationally [11,12] as are musculoskeletal studies specifically demonstrating the utility of case-crossover designs to examine transient physical and psychological triggers of low back pain [13-15], knee OA [16-18], and hip OA [19]. Although flare-ups appear to be real phenomena experienced by people with knee OA [11], the antecedents that cause flare-ups and their consequences remain unclear. Our hypothesis is that although susceptibility to flares may be determined by a range of factors, they are ultimately triggered by short-term exposures. The etiopathogenesis of OA is believed to reflect a joint’s long-term attempt to accommodate or regulate cumulative excessive or aberrant loading [20]. We postulate that intermittent or transient activity–related exposures that precipitate short-lived recurrent painful flare-ups are key to this process and related management.

The primary objective of this study is to identify the most common and consistently associated proximate causes (*triggers*) of knee OA flares to help make acute flares more predictable and therefore potentially preventable. Secondary objectives are to

1. estimate the time course of acute flares in knee OA with regard to symptoms, activities, and role interference to provide better information to patients and practitioners on the likely short-term prognosis.
2. determine whether characteristics of the participant and his or her problem can identify individuals who are susceptible to flares (*frequent flare phenotypes*) to target flare management and preventive advice in practice.

## Methods

### Study Design

This study is a Web-based case-crossover study [21]. This self-controlled design assembles within-person case-control comparisons to establish if transient or intermittent exposures (potential triggers) before acute or abrupt-onset events (knee OA flare-up) may explain these episodes. Case-crossover designs have been used to investigate triggers of acute-onset disease (eg, myocardial infarction [22] and stroke [23]), health care events (eg, [24,25]), and acute-on-chronic episodes (eg, gout flares [26]). In this study, the case-crossover design was chosen as an efficient method to identify recurrent *acute-on-chronic* events [27], while capturing proximate exposures. This design is particularly valuable in the context of triggers of acute flares because it controls for time invariant confounders, under the assumption that there are no time trends in exposures over the period of investigation [28]. By conducting this study using a Web-based platform, data collection is efficient in terms of time and cost, while also enabling capture of real-time information on recurrent flares [29].

### Target Population

Community-dwelling adults aged 40 years and over, who are resident in England, with knee pain and/or knee OA and have daily access to email and the internet will be invited to take part in this study. A full list of eligibility criteria is presented in Table 1.

**Table 1:** Eligibility criteria.

Eligibility criteria		Mode of ascertainment
**Inclusion criterion**		
	Male or female aged ≥40 years, resident in England	GPSS^a^ or registration page
	Registered as a permanent resident with participating general practices^b^	GPSS
	Consultation for knee OA or knee OA-related joint symptoms in the last 2 years^b,c^	GPSS
	Daily access to an email account and to the internet (laptop, desktop, tablet, or smartphone)	PCRF^d^ or registration page
**Exclusion criterion**		
	Known diagnosis of inflammatory arthropathy, spondyloarthropathy or crystal arthropathy (eg, rheumatoid arthritis, ankylosing spondylitis, reactive arthritis, systemic lupus erythematosus, gout, and psoriatic arthritis), fibromyalgia^c^	GPSS or registration page
	Symptoms are from a knee that has been replaced	GPSS or registration page
	Surgery to either knee within the past 3 months	GPSS or registration page
	Unable to complete questions written in English	PCRF or registration page
	Vulnerable individuals (eg, psychiatric illness, learning difficulties, dementia, terminal illness, and severe enduring mental ill health)^b^	GPSS

^a^GPSS: General practice search and screen.

^b^Applicable only to participants recruited via general practices.

^c^Based on code lists (available upon request).

^d^PCRF: patient-completed reply form.

### Recruitment Procedures

Recruitment will be done via 3 routes: (1) general practice registers, (2) offline community advertisement, and (3) online community advertisement.

#### General Practice Registers

In total, 5000 potentially eligible adults with suspected or diagnosed clinical knee OA will be identified from up to 17 general practices across England and will be mailed a study pack (letter of invitation, Participant Information Sheet (PIS), reply form, and prepaid return envelope). General practitioners at each practice will also be invited to screen the sample list for patients whom they consider should be excluded from the invitation mailing (eg, vulnerable individuals). A reminder letter, together with a repeat PIS, reply form, and prepaid return envelope, will be sent to people who have not responded after 2 weeks. People who have not responded within 4 weeks will not be contacted again. Practice mailing will be performed in stages.

Participants who return a reply form will be providing implied consent to further contact. Those who, on their reply form, fulfill the eligibility criteria and provide their name, a valid personal email address, and mobile phone number (optional) will be sent a preconsent welcome email containing a Web link to the ACT-FLARE study website (developed using Microsoft Visual Studio). The Web link will direct them to an online copy of the PIS, and from there to the informed electronic consent (e-consent) form. For people who return a reply form with illegible, ambiguous, or invalid responses or personal details (eg, invalid email address), an attempt to clarify this information will be made with 1 follow-up email or letter, as appropriate, asking the individual to contact the study team by phone or email. If there is no response, these people will not be contacted again.

#### Offline Community Advertisement

Study posters, flyers, or business card advertisements will be displayed in the waiting areas and patient information points of all participating general practices; additional general practices; selected community pharmacies; patient waiting areas in community hospitals; and public libraries across England, where permission to do so is granted. Wherever possible, the study will also be publicized through local newspapers and radio.

The advertising material will include the study title, summary study information and eligibility, the Web address for the ACT-FLARE study registration webpage, and study contact email address and telephone number. Members of the public who are interested in taking part and believe they may be eligible will be invited to visit the ACT-FLARE study registration webpage where summary information, eligibility, and contact details are again displayed. Interested members of the public who deem themselves eligible will be asked to submit key data (implied confirmation of eligibility, email address, name (optional), mobile phone number (optional), postcode (to ensure they are an England resident), and how they heard about the study) to Keele Clinical Trials Unit, which will generate a unique study identification number and the preconsent welcome email containing a Web link to the ACT-FLARE study website. The Web link will direct them to a copy of the PIS and to the informed e-consent process.

#### Online Community Advertisement

We will use targeted social media advertising in Facebook to publicize the ACT-FLARE study. This will also include placement of adverts on selected key organizational or group pages, such as Arthritis Research UK, Arthritis Care, Age UK, and Patient UK, as well as Keele University Research Institute for Primary Care & Health Sciences website, Facebook page, Twitter account, and blog. Interested, potentially eligible participants will be directed to the ACT-FLARE study registration page where the same subsequent process of recruitment as described in the offline route will be followed.

All participants will be invited to provide informed e-consent before setting up a username and password for login access to the ACT-FLARE study website to participate in the study.

Ethical approval has been obtained from Yorkshire & The Humber—Leeds East Research Ethics Committee (REC reference number: 18/YH/0075).

### Data Collection

Data will be collected via self-complete questionnaires by the participant using a Web-based data collection platform previously developed as part of our feasibility and pilot study [30]. All participants will be followed up for a 13-week period, irrespective of how many flare-up episodes are experienced. Data collection will comprise 4 elements: (1) baseline questionnaire, (2) scheduled questionnaires, (3) event-driven questionnaires, and (4) daily questionnaires during a flare-up. Data collection has the following general features:

1. All questionnaires can be completed on a desktop, laptop, tablet, or mobile smartphone in under 15 min, in accordance with feedback from our Patient Advisory Group (PAG).
2. Questionnaires must be completed in one time point by the participant. There is no facility for partial completion and return at a later time. This approach was selected to ensure that questionnaires are completed as contemporaneously as possible.
3. Once a questionnaire has been completed and submitted by a participant through the website, the participant no longer has repeat access to the questionnaire.
4. A short onscreen *thank you* statement will be generated following the completion of each questionnaire.
5. Remembering to notify the research team about a flare-up has been identified as a critical issue. Participants who provide a mobile telephone number (optional) will be sent a reminder text message about the study once a fortnight for the duration of the study. This will be calculated from the date each participant completes their baseline questionnaire, and all texts will be sent to participants at 18:00 Greenwich Mean Time.
6. At the end of the study, participants will be invited to provide feedback on their participation in the study and whether they would be willing to additionally participate in clinical examinations during a knee flare-up, should a similar study be conducted in the future, to include magnetic resonance imaging and synovial fluid sample via knee joint aspiration.

### Baseline Questionnaire

The purpose of the baseline questionnaire is to provide descriptive information on participants’ history of knee pain and flare-ups, current knee features, health care use for knee pain, general health, including normal physical activity exposures, and demographics. The content of the baseline questionnaire is provided in Table 2. It includes domains and measurement instruments of potential relevance based on previous literature and critical input from a PAG.

Once participants activate their log-in with the study website, they will be directed to the baseline questionnaire, which becomes available for completion immediately. At this point, participants will also receive emailed instructions to use their username and password to log-in to the website. If participants do not complete the baseline questionnaire, an email reminder will be sent after 3 days and a repeat email reminder after a further 3 days. If no response is received after 7 days from the initial date of invitation, participants can no longer continue in the study. The participant will receive an email notification confirming this.

**Table 2:** Baseline questionnaire.

Concept, measurement method			Detail	Time available for completion	Reminder sent
**Section A: Your knee pain**					
	Time since onset		Not applicable, <1 year, 1-4 years, 5-9 years, 10+ years. Left, right	7 days	Yes
	Pattern [31]		5 flare pattern illustrations. Left, right	7 days	Yes
	Experience of knee pain [32]		In past 6 months: No pain, predictable pain, some unpredictability, constant. Left, right	7 days	Yes
	Pain, aching, stiffness in last month [33,34]		No days, few days, some days, most days, all days. Left, right	7 days	Yes
	Worst and least in last week, average, current [35]		0-10 Numerical Rating Scale with anchors (no pain, pain as bad as you can imagine)	7 days	Yes
	Knee injury and Osteoarthritis Outcome Score Physical Function [36]		7-items and 5-option categories for difficulties with daily activities in last week	7 days	Yes
	Knee injury and Osteoarthritis Outcome Score Quality of Life [37]		4-items and 5-option categories for quality of life in last week	7 days	Yes
	Bothersomeness in last 24 hours [38]		Not at all, slightly, moderately, very much, extremely. Left, right	7 days	Yes
	Flare-up at present		Yes, No. Left, right	7 days	Yes
	Self-reported main flare trigger		Free text	7 days	Yes
	Varus-valgus malalignment [39]		Very bow legged, bow legged, normal, knock-knee, very knock-knee. Left, right	7 days	Yes
	Foot rotation [39]		Very turned out feet, turned out feet, straight, turned in feet, very turned in feet. Left, right	7 days	Yes
	Previous knee injury [40]		Injury induced walking problems for at least 1 week. Left, right	7 days	Yes
	Family history of total knee replacement [40]		Mother, father, sister or brother. Yes, No, don’t know	7 days	Yes
**Section B: Health care use for your knee pain**					
	Medications, last week		17-option categories for drug use, tick as many boxes as apply	7 days	Yes
	Health professional consultation, last year		General practitioner, practice or district nurse, physiotherapist, surgeon, rheumatologist, acupuncturist, occupational therapist	7 days	Yes
	Any kind of previous knee surgery		Yes, No. Left, right	7 days	Yes
	Previous knee injections last 3 months		Left, right, both, not applicable	7 days	Yes
	Previous knee surgery last 3 months [40]		Left, right, both, not applicable	7 days	Yes
	Previous total knee replacement		Left, right, both, not applicable	7 days	Yes
	On waiting list for total knee replacement		Left, right, both, not applicable	7 days	Yes
**Section C: Your general health**					
	Perceived general health [41]		Excellent, very good, good, fair, poor	7 days	Yes
	Physical activity [42]		Work physical activity (5-response options), general physical activity in last week (5-response options, 4-option categories), walking pace	7 days	Yes
	Self-reported weight		Stones and lbs or kg	7 days	Yes
	Self-reported height		Feet and Inches or cm	7 days	Yes
	**Normal physical activities on a normal day**				
		Walking outside without rest	Not at all, A little, A lot	7 days	Yes
		Standing for long periods without rest	Not at all, A little, A lot	7 days	Yes
		Sitting for long periods without a break	Not at all, A little, A lot	7 days	Yes
		Moderate-to-vigorous physical activity (this may include activities that make you breath harder than normal) [43]	Not at all, A little, A lot	7 days	Yes
		Going up and down stairs	Not at all, A little, A lot	7 days	Yes
		Driving	Not at all, A little, A lot	7 days	Yes
		Squatting or kneeling	Not at all, A little, A lot	7 days	Yes
		Lifting or moving heavy objects	Not at all, A little, A lot	7 days	Yes
		Going up and down ladders	Not at all, A little, A lot	7 days	Yes
**Section D: About you and your circumstances**					
	Gender		Male, female	7 days	Yes
	Date of birth		Date, Month, Year	7 days	Yes
	Current employment		Paid employment or self-employed, retired, looking after home and/or family, unable to work because of sickness or disability, unemployed, doing unpaid or voluntary work, full or part-time student.	7 days	Yes

### Scheduled Questionnaires

The purpose of the scheduled questionnaires is to measure activities and exposures (potential triggers) during control periods (ie, days that are not followed by a flare-up). The content for the scheduled questionnaire is provided in Table 3. It features a matrix for reporting the occurrence and amount of 21 physical, psychosocial, and environmental potential triggers on the day of questionnaire completion and the 3 days before this. These potential triggers have been selected from previous literature, PAG discussion, and clinical experience.

In total, 4 scheduled questionnaires will be sent to participants 1 week, 5 weeks, 9 weeks, and 13 weeks after completion of the baseline questionnaire. The timing of all scheduled questionnaires remains the same in the event of delayed or nonresponse to 1 or more scheduled questionnaires. All participants will be sent an email inviting them to complete each scheduled questionnaire about their activities and exposures during the last 3 days. The email will contain a direct link to the questionnaire, which will also become accessible at the correct point in time should the participant log-in to the website independently of the email link.

As a part of each scheduled questionnaire, participants will initially be asked if they are currently experiencing a flare-up of their knee pain. If *no*, they will continue to complete the scheduled questionnaire. If *yes*, the participant will be redirected to complete the event-driven questionnaire (see below).

Nonresponders will be sent an email reminder after 3 days and a repeat email reminder after a further 3 days. If no response is received after 7 days, the questionnaire becomes deactivated and can no longer be completed. The participant will receive an email notification confirming this. Nonrespondents to scheduled questionnaires remain in the study.

### Event-Driven Questionnaires

The purpose of the event-driven questionnaires is to measure activities and exposures (potential triggers) during hazard periods (ie, the 3 days before experiencing a flare-up) and to gauge whether flare-ups are often anticipated by participants. The content for the event-driven questionnaire is provided in Table 4. As per the scheduled questionnaire, this features the same matrix for reporting the occurrence and amount of 21 physical, psychosocial, and environmental potential triggers on the day the flare-up started and the 3 days before this. In addition, participants will be asked about features of their flare-up.

Participants will be invited to complete an event-driven questionnaire immediately if they provide notification through the website that they are currently experiencing a self-reported flare-up. This notification can be initiated either via a Web link provided in the welcome email, scheduled questionnaire email, text message correspondence, or by logging onto the study website. There is no limit to the number of times a participant can self-report a flare-up episode during the study period.

After providing a flare-up notification, if a participant does not complete the event-driven questionnaire, an email reminder will be sent after 1 day. A repeat email reminder will be sent after a further day. If no response is received after 2 days, the event-driven questionnaire becomes deactivated and can no longer be completed. The participant will receive an email notification confirming this. Nonrespondents to event-driven questionnaires remain in the study.

**Table 3:** Scheduled questionnaire. Potential trigger questions answered for today, day before, 2 days earlier, and 3 days earlier.

Concept^a^, measurement method			Detail	Time available for completion	Reminder sent
**Knee pain**					
	Flare-up at present		Yes, No. Left, right	7 days	Yes
	Average pain in last 24 hours [44]		0-10 Numerical Rating Scale with anchors (no pain, pain as bad as you can imagine). Left, right	7 days	Yes
**Potential triggers**					
	**Physical activities**				
		Walking outside without rest	Not at all, A little, A lot	7 days	Yes
		Standing for long periods without rest	Not at all, A little, A lot	7 days	Yes
		Sitting for long periods without a break	Not at all, A little, A lot	7 days	Yes
		Moderate-to-vigorous physical activity (this may include activities that make you breath harder than normal) [43]	Not at all, A little, A lot	7 days	Yes
		Going up and down stairs	Not at all, A little, A lot	7 days	Yes
		Driving	Not at all, A little, A lot	7 days	Yes
		Squatting or kneeling	Not at all, A little, A lot	7 days	Yes
		Lifting or moving heavy objects	Not at all, A little, A lot	7 days	Yes
		Going up and down ladders	Not at all, A little, A lot	7 days	Yes
	**Slips, trips, sprains, and strains**				
		Slip, trip or fall	No, Yes	7 days	Yes
		Episode of buckling or giving way [45]	No, Yes	7 days	Yes
	**Health and health care use**				
		Reduce or miss medication	No, Yes	7 days	Yes
		Take extra medication	No, Yes	7 days	Yes
		Cough, cold, or other minor infection	No, Yes	7 days	Yes
	**Stress and other things**				
		Work-related stress [46]	No, Yes	7 days	Yes
		Home-related stress [46]	No, Yes	7 days	Yes
		Friend/family-related stress [46]	No, Yes	7 days	Yes
		Low mood/depression	No, Yes	7 days	Yes
		Feeling angry, irritable, or hostile	No, Yes	7 days	Yes
		Poor night’s sleep	No, Yes	7 days	Yes
		Generally cold and damp weather [47]	No, Yes	7 days	Yes

^a^Questionnaire opens with orientation text: The following questions are about your knee symptoms at the moment. Please answer all questions below. Some of these questions will ask you about things you may have been doing on the last 3 days. Please can you take a moment to remind yourself what you were doing on each of these days to help you answer some of the questions below.

**Table 4:** Event-driven questionnaire. Potential trigger questions answered for day of flare-up, day before, 2 days earlier, and 3 days earlier.

Concept^a^, measurement method			Detail	Time available for completion	Reminder sent
**Nature of flare**					
	**Knee pain**				
		When did this flare-up start?	Today, yesterday, 2 days ago, 3 days ago, 4 or more days ago	2 days	Yes
		Was this flare-up unexpected?	Yes, No	2 days	Yes
		Which knee currently experiencing a flare-up?	Left, right	2 days	Yes
		Average pain in last 24 hours [44]	0-10 Numerical Rating Scale with anchors (no pain, pain as bad as you can imagine). Left, right	2 days	Yes
	**Changes noticed since flare-up**				
		More than usual: limping, swelling, stiffness, increased difficulty with activities of daily living, sleep disturbance by knee pain	Tick as many boxes as apply	2 days	Yes
**Potential triggers**					
	**Physical activities**				
		Walking outside without rest	Not at all, A little, A lot	2 days	Yes
		Standing for long periods without rest	Not at all, A little, A lot	2 days	Yes
		Sitting for long periods without a break	Not at all, A little, A lot	2 days	Yes
		Moderate-to-vigorous physical activity (this may include activities that make you breath harder than normal) [43]	Not at all, A little, A lot	2 days	Yes
		Going up and down stairs	Not at all, A little, A lot	2 days	Yes
		Driving	Not at all, A little, A lot	2 days	Yes
		Squatting or kneeling	Not at all, A little, A lot	2 days	Yes
		Lifting or moving heavy objects	Not at all, A little, A lot	2 days	Yes
		Going up and down ladders	Not at all, A little, A lot	2 days	Yes
	**Slips, trips, sprains, and strains**				
		Slip, trip, or fall	No, Yes	2 days	Yes
		Episode of buckling or giving way [45]	No, Yes	2 days	Yes
	**Health and health care use**				
		Reduce or miss medication	No, Yes	2 days	Yes
		Take extra medication	No, Yes	2 days	Yes
		Cough, cold, or other minor infection	No, Yes	2 days	Yes
	**Stress and other things**				
		Work-related stress [46]	No, Yes	2 days	Yes
		Home-related stress [46]	No, Yes	2 days	Yes
		Friend/family-related stress [46]	No, Yes	2 days	Yes
		Low mood/depression	No, Yes	2 days	Yes
		Feeling angry, irritable, or hostile	No, Yes	2 days	Yes
		Poor night’s sleep	No, Yes	2 days	Yes
		Generally cold and damp weather [47]	No, Yes	2 days	Yes
**Your flare-up**					
		What do you think caused this flare-up of your knee pain?	Free text	2 days	Yes

^a^Questionnaire opens with orientation text: The following questions are about your current flare-up of knee pain. Please answer all questions below. Some of these questions will ask you about things you may have been doing on the 3 days before your flare-up and also on the day it started. Please can you take a moment to remind yourself what you were doing on each of these days to help you answer some of the questions below.

### Daily Questionnaires During a Flare-Up

The purpose of the daily questionnaire during a flare-up is to collect information regarding the natural course of flare-up episodes. These comprise 4 brief questions on pain intensity, bothersomeness, health care use, and participant judgment on whether their flare-up has ended. The content for the daily questions during flare-up is provided in Table 5.

Upon completion of the event-driven questionnaire, participants will be invited to complete the 4 questions, starting one day later, via email until resolution of their flare-up. *Resolution* is defined as participants reporting that their symptoms have returned to their preflare *normal* state for 2 consecutive days.

There will be no reminders for the daily questionnaires during flare-up and participants can only complete questionnaires on the day they are sent, with any earlier incomplete dates becoming deactivated. Emails will be sent to participants at 18:00 Greenwich Mean Time for the duration of the flare-up episode. Broderick et al [48] have previously demonstrated that end-of-day pain measurement adequately reflects average daily pain levels. If a participant is still reporting that they are in a flare-up episode at the end of the study period, they will not be followed up to flare-up resolution beyond this time point. If a participant is having a flare-up, they will not receive a further scheduled questionnaire until the participant notifies us that the flare-up episode has resolved.

**Table 5:** Daily questions during a flare-up.

Concept, measurement method		Detail	Time available for completion	Reminder sent
**Knee pain**				
	Average pain in last 24 hours [44]	0-10 Numerical Rating Scale with anchors (no pain, pain as bad as you can imagine). Left, right	6 hours	No
**Impact of pain**				
	Bothersomeness in last 24 hours [38]	Not at all, slightly, moderately, very much, extremely. Left, right	6 hours	No
**Medication use**				
	Pain medication taken in last 24 hours	No; yes, but less than usual; yes, about the same as usual; yes, more than usual	6 hours	No
**Flare-up resolution**				
	Has your flare-up ended	Yes, No	6 hours	No

### Patient Involvement

This study is a patient-confirmed research priority, and a study-specific PAG has assisted with the study development from inception. So far, this has included advice and suggestion on all aspects of questionnaire and website development as part of this full-scale study and our previous feasibility and pilot study [30]. Engagement has taken place through workshops, written and verbal feedback on study questionnaires, and practical hands-on trial of website utility during development. One member of our PAG has remained an active member of the study management group from inception (CP).

### Outcome Definition

Self-reported flare-up of symptomatic knee OA is defined as “an event in the natural course of the condition characterized by a change in the participant’s baseline pain that is beyond normal day-to-day variation, sustained for at least 24 hours, and is sudden or quick in onset. It may impact on the ability to perform everyday activities and result in an increase in analgesic intake”. This definition was derived from our pilot study [30], which used a qualitative approach based on self-assessment, a previous literature review [49], group discussions with patients and members of the public, and findings from a previous survey and 3-month pen-and-paper daily diary study (unpublished data at time of submission).

### Sample Size

A sample size of 434 participants will have 80% power at a 5% 2-tailed significance level to detect an unadjusted odds ratio of 2 for knee pain flare-up in the hazard period relative to control period if the probability of exposure (potential trigger) among control periods is at least 0.1, the correlation coefficient for the exposure between matched hazard periods and control periods is no more than 0.3 [16], and assuming a 1:1 ratio of control periods to hazard periods.

We will recruit 620 participants, allowing for approximately 30% of participants who may not experience a flare-up or drop-out during the study period [16]. We estimate a total of 17 general practices will be required for this recruitment target. This is based on 8% of adults aged ≥40 years consulting for knee OA or knee joint pain over a 2-year period [50], an average practice list size of 7000 patients, half of whom are aged ≥40 years, and a combined eligibility, response, and consent rate of 12%.

Recruitment of participants via offline and online community advertisements will efficiently supplement the above recruitment method, which will be particularly valuable in the event of lower-than-expected participation and reported flare frequency, and for reducing imprecision in important subgroup analyses, for example, restricting analysis to participants who provide early notification of flare-up and those whose flare-up proves to be more than transient.

### Statistical Analysis

#### Summary of Baseline Data and Flow of Participants

##### Recruitment and Retention

Production of a Consolidation Standards of Reporting Trials–style participant flowchart and simple descriptive statistics for response rates (including age, gender, and deprivation score, derived from participant postcodes, of responders compared with eligible nonresponders at baseline) in accordance with standard definitions [51].

##### Summary Descriptive Characteristics of the Study Sample

Demographic and self-report clinical characteristics will be described. Participants will be compared with ineligible and nonconsenting participants on available data. Summary descriptive characteristics of flare-ups, symptoms, and consequences during the flare-up will be described.

#### Triggers, Course, Consequences, and High-Risk Participant Profiles

##### Proximate Triggers of Acute Flares (Primary Objective)

The odds of identified potential flare-up antecedents or triggers occurring in the hazard period will be compared with the relative occurrence in the control period using conditional logistic regression using m:n matching, as each participant may have multiple hazard and control periods [52]. Modeled data will be presented as odds ratios with 95% CIs. The assumption of no time-trend in exposure will be verified.

The optimal duration of the hazard (effect) period for flare-ups is unknown. Our primary analysis will be based on the scheduled questionnaires (Weeks 1, 5, 9, and 13) being the main source of control exposure measurements comparison for with the event-driven questionnaire. The relative merits of hazard periods of 24, 48, and 72 hours will be explored to test the induction period, while also protecting the analysis against rare exposures.

In the advent of low levels of completion of the scheduled questionnaire, we will explore alternative sources of control measurement: (1) normal physical activity exposure measurements ascertained in the baseline questionnaire and (2) by exposure measurement in the preceding 48 and 72 hours (for hazard period exposure defined in the preceding 24 hours) within the event-driven questionnaire.

Owing to a lack of consensus definition for a flare-up in the OA literature, our statistical analysis plan will allow for analysis of alternative definitions, for example, no defined minimum flare-up duration, or imposed knee pain change score of ≥2 on a numerical rating scale, between baseline and self-reported flare-up. We will also consider the potential for combining related exposures.

By describing the proportion of triggers during the hazard window that were reported by participants as being unanticipated, the extent to which triggers were predictable will be explored.

##### Time Course and Consequences of Acute Flares (Secondary Objective 1)

Using the daily questionnaires during a flare-up, time-to-resolution of symptoms will be compared across participants.

##### Frequency of Acute Flares (Secondary Objective 2)

The rate of acute flares reported during follow-up will be modeled using regression models. This will identify whether certain participants and flare characteristics (collected in the baseline questionnaires) are more or less likely to experience flare-ups.

The amount of missing data will be calculated and the effects on each of the analyses may be investigated using multiple imputation.

## Results

Participant recruitment opened in July 2018 and is anticipated to continue for 6 months. The study results will be disseminated through a number of channels, including relevant national or international conferences and peer-reviewed publication in a medical journal, via advocacy or charity organizations, such as Versus Arthritis and across social media. Findings will be fed back to members of our PAG, study participants, and clinicians from participating primary care general practices. The PAG will also take an active role in the overall dissemination strategy.

## Discussion

Recognition of the potential importance of episodic flares in the natural history of OA is gaining momentum among the clinical research community [49,53,54]. In this 13-week Web-based case-crossover study, we will combine general practice–based recruitment with social media advertising across England to identify proximate causes (*triggers*) of acute flare-ups in knee OA, estimate their time course and consequences, and describe individuals most susceptible to flares.

By embracing both digital epidemiology and within-person study design to examine OA flares, it is hoped that real-time observations of individual episodic symptom variability can provide insights into these phenomena and their potential relation to short-term prognosis. However, this endeavor is not without limitations. Major challenges of this approach are the recruitment of individuals to a Web-based data collection platform and timely capture of events and exposures. With no agreed objective measurement for an OA flare, the ascertainment of onset is reliant on participant self-report. There is also the potential for recall bias owing to differential reporting of exposure in the hazard and control periods. For example, participants answering questions about potential exposures over the last 3 days while currently experiencing a flare-up (event-driven questionnaire) may respond differently to the same questions when they are not experiencing a flare-up (scheduled questionnaire). Despite this, all comparisons are within-person, therefore eliminating time-invariant person-level confounders by design. Conditional regression is then used to compare exposure status between the hazard and control periods within the same person. Factors that do not change over time, such as gender and genetics remain constant in all periods. In addition, inviting participants to reflect on recent experiences to help understand the behavior of their symptoms can be easily integrated into patient-oriented approaches to self-management that can occur in the community and be supported by all primary care encounters. This study will provide empirical evidence to help patients identify common knee OA flare triggers, provide health care professionals with questions to identify patients at most risk of frequent flare-ups, and inform clinical guidelines.

## Abbreviations

CRN: clinical research network
e-consent: electronic consent
HEE: Health Education England
NIHR: National Institute for Health Research
OA: osteoarthritis
PAG: Patient Advisory Group
PIS: Participant Information Sheet
